# Acute toxicity of TNT derivatives and hydrazine-based compounds from explosive and rocket fuel contamination to darkling beetles (*Tenebrio molitor* and *Opatrum sabulosum*)

**DOI:** 10.7717/peerj.20427

**Published:** 2026-02-05

**Authors:** Denis F. Rybalka, Viktor V. Brygadyrenko

**Affiliations:** 1 Department of Biodiversity and Ecology, Oles Honchar Dnipro National University, Dnipro, Ukraine; 2 Dnipro State Agrarian and Economic University, Dnipro, Ukraine

**Keywords:** Coleoptera, Tenebrionidae, *Tenebrio molitor*, *Opatrum sabulosum*, LC50, TNT, HMX, RDX, UDMH, Explosives

## Abstract

The problem of toxic pollution of the environment by combustion products of explosives and rocket fuel is becoming increasingly important in the context of intensified military operations. In this study, the toxicity of TNT derivatives, hydrazine-based compounds, and nitroaromatic transformation products related to explosive and rocket fuel contamination was evaluated using the model organisms *Tenebrio molitor* Linnaeus, 1758 and *Opatrum sabulosum* (Linnaeus, 1761). We chose hydrazine derivatives, nitroanilines, benzene and naphthalene-based nitro compounds that can persist in the ground, water and atmosphere after explosions or incomplete combustion of rocket fuel. Topical dispersion was used to evaluate exposure of specified doses on the surface of containers containing larvae and imagoes of *T. molitor* and imagoes of *O. sabulosum*, followed by lethality counts and LC_50_ determination. The results indicate the high sensitivity of *T. molitor* larvae and imagoes and *O. sabulosum* imagoes to TNT derivatives, hydrazine compounds, and nitroaromatic contaminants commonly found in explosive-contaminated environments, which confirms the feasibility of introducing ecotoxicological monitoring of military-technological impact zones. The systematic toxicity assessment of these 29 explosive-related compounds provides essential baseline data for environmental risk modeling and ecotoxicological monitoring programs. The data obtained can be used for further modeling of environmental risk and development of bioindicator approaches to detect pollution as a result of military operations, for example, in Ukraine.

## Introduction

Full-scale military operations, ammunition testing, and regular missile launches cause widespread environmental pollution with toxic transformation products and explosive derivatives, including TNT derivatives, hydrazine-based compounds, and nitroaromatic contaminants related to explosive and rocket fuel systems. These transformation products and environmental derivatives enter the soil, water bodies, and atmosphere, where they can persist for a long time, affecting microbial communities, vegetation, and animals (Leal Filho et al., 2024; Solokha et al., 2024). Studies show that explosive derivatives and rocket fuel transformation products are highly resistant to natural degradation, accumulate in ecosystems, and are detected in soils and groundwater near training ranges and combat zones (US Army Corps of Engineers et al., 2005; Yang et al., 2023). The environmental fate and transport of energetic materials involves complex interactions with soil minerals and water systems, where compounds like TNT can persist through specific adsorption mechanisms that affect their bioavailability and degradation pathways (Hurley & Paul, 2009). According to environmental monitoring data, explosive-related derivatives and transformation products are common pollutants released into the environment during military operations and negatively affect soil biocenoses, including invertebrate diversity (Broomandi et al., 2020). The ongoing conflict in Ukraine since 2022 has dramatically increased the urgency for systematic assessment of explosive-related contamination, as widespread military activities generate extensive environmental pollution requiring immediate toxicological evaluation. These compounds pose particular risks in conflict zones where persistent contamination affects both civilian populations and ecosystems for years after active combat ceases.

Among the compounds formed as a result of the combustion of rocket fuel or used as components of explosives, a significant part are derivatives of nitroaromatic and hydrazine compounds. These include p-nitroaniline, m-nitroaniline, 2-methyl-5-nitroaniline, 2,4-dinitroaniline, 1-methyl-2,4-dinitrobenzene, and hydrazine hydrate. These substances are highly toxic even at low concentrations, with the ability to disrupt metabolism, inhibit enzyme activity, and affect the behavioral reactions of invertebrates, including insects (US Army Corps of Engineers et al., 2005; Yang et al., 2023; Corredor et al., 2024). The toxicity of these compounds, particularly TNT derivatives, involves metabolic activation pathways that generate reactive oxygen species (ROS) and cause oxidative stress, leading to cellular damage and macromolecular dysfunction (Adomako-Bonsu, Jacobsen & Maser, 2024). Recent studies have demonstrated that TNT undergoes reductive metabolic activation, producing nitroso and hydroxylamine intermediates that interact with cellular components to cause DNA and protein adduct formation (Adomako-Bonsu, Jacobsen & Maser, 2024). Despite the relative availability of these compounds in pesticides, fuel mixtures, or munitions, data on their ecotoxicological effects on terrestrial invertebrates remain limited, especially in the context of chronic or sublethal exposure (Corredor et al., 2024). Studies on *Tenebrio molitor* Linnaeus, 1758 have already noted that organic toxicants, including neurotoxins, can cause mortality, weight loss, behavioral changes, and reduced life span, which makes this species a reliable test organism in toxicological studies (Brai et al., 2023).

The use of *Tenebrio molitor* and and *Opatrum sabulosum* (Linnaeus, 1761) is justified by their ecological relevance in explosive-contaminated environments, making them suitable models for environmental risk assessment in military-impacted areas (Brai et al., 2023). According to the “3Rs” (replacement, reduction, refinement) principles, which have been in effect since 1959, toxicologists are obliged to reduce the use of vertebrates if alternatives exist. Given this, insects have become an attractive model for the initial determination of *in vivo* toxicity: they are cheap to rear, not subject to strict legal regulation, easy to maintain even for untrained staff, and are suitable for statistically valid ecotoxicological short-term experiments (Brai et al., 2023). While only limited comparative studies exist between *T. molitor* and mammalian models, *T. molitor* has demonstrated potential as a cost-effective preliminary screening tool that can bridge *in vitro* and mammalian studies for specific compound classes (Brai et al., 2023).

For the study, we selected 29 organic compounds that are derivatives, transformation products, or environmental contaminants related to explosive and rocket fuel compounds. Our selection focused specifically on TNT derivatives (such as dinitrotoluenes and nitroanilines), hydrazine-based compounds (including hydrazine hydrate and dinitrophenylhydrazine), and nitroaromatic compounds commonly found in explosive-contaminated sites. While TNT has been widely used in munitions and military systems, our study targeted environmental transformation products and structurally related compounds that persist in contaminated environments through incomplete combustion, munitions disposal, and contamination from manufacturing sites. In the environment, they are detected as xenobiotic contaminants of soils, water bodies and atmospheric dust in the territories of military training grounds and conflict zones, where they persist for years due to their resistance to decomposition. Derivatives of nitroanilines, chlorinated benzene and nitrophenols are found among toxic residues after fuel combustion, explosions and chemical transformation of explosives, which confirms the feasibility of their inclusion in the list of substances for ecotoxicological monitoring (US Army Corps of Engineers et al., 2005; Broomandi et al., 2020). This allows representative coverage of the main groups of pollutants generated during military operations, air strikes, fuel combustion, or ammunition destruction.

Establishing the level of toxicity has an applied purpose—to identify those compounds that are the most dangerous and difficult to biodegrade, which is critical for the development of bioremediation technologies. Current approaches to biological cleanup of soil and water from explosive and rocket fuel residues include microbial consortia, enzymatic methods, electrokinetic remediation, and even nanotechnology (Chatterjee et al., 2017; Kalsi et al., 2020). The degradation kinetics of hydrazine compounds involve complex oxidative reactions that must be understood for effective remediation strategies (Fu et al., 2022). The effectiveness of these methods depends not only on the presence of appropriate microorganisms, but also on the nature and bioavailability of toxicants. Assessment of the toxicity of organic explosive compounds in model species (such as *T. molitor* and *O. sabulosum*) can provide important information on the persistence of contamination in conflict zones or near military training areas where chemical cleanup is technically difficult or economically unfeasible (Singh et al., 2019; Binder et al., 2025). Recent advances in marine invertebrate models have demonstrated effective approaches for studying explosive compound depuration and bioaccumulation patterns, providing complementary insights to terrestrial invertebrate toxicology (Binder et al., 2025).

We investigated the acute toxicity of explosives, rocket fuel, and their transformation products to invertebrates, specifically larvae and imagoes of *T. molitor* and imagoes of *O. sabulosum*, through surface contact (ingestion through the cuticle and tracheal system of insects rather than the intestine). The aim of this study is to establish the acute toxicity (LC_50_) of 29 organic compounds associated with explosives and rocket fuel for larvae and imagoes of *T. molitor* and imagoes of *O. sabulosum*.

## Materials and Methods

### Solvent analysis

In order to control the background toxicity of the solvents we conducted separate experiments for ethanol, dimethyl sulfoxide (DMSO) and saline (0.9% NaCl solution in water). The solutions were applied superficially by spraying 0.36 mL on a 10 × 10 cm (0.01 m^2^) container, which corresponds to the exposure area in the main experiment. We tested all containers for 24 h to detect possible mortality or changes in the behavior of the experimental organisms. Throughout the experimental trials, we identified safe concentrations of solvents that did not cause mortality or behavioral changes in *T. molitor* and *O. sabulosum*.

As expected, saline did not cause any deaths. Ethanol at a concentration of 96 g/m^2^ caused the death of half of the experimental organisms, and no increase in mortality was observed at a concentration of 24 g/m^2^or less (Table 1). Testing of DMSO at nine doses in the range of 0.0625–4.00 g/m^2^ allowed us to determine the acute toxicity (LC_50_), which was 0.964 ± 0.196 g/m^2^ (mean ± SE). Despite the detected toxicity, the use of DMSO was necessary because a number of the studied substances are insoluble in ethanol or saline. DMSO ensured the stability and homogeneity of the solutions, which was important for the accuracy of dosing in our experiment. We used control groups (Table 2) with the appropriate concentration of DMSO to distinguish between the effects of the solvent and the test substance.

**Table 1:** Mortality in 24 h of *T. molitor* larvae in one day from exposure to different concentrations of ethanol, studied for the purpose of using this solvent (*n* = 6).

Ethanol, g/m^2^	16	24	48	96	192
Survival rate, %	100.0	100.0	75.0	50.0	0.0

**Table 2:** Mortality in 24 h of *T. molitor* larvae exposed to different concentrations of dimethyl sulfoxide (DMSO), investigated for use as a solvent (*n* = 6; LC_50_= 0.964 ± 0.196 g/m^2^, mean ± SE).

DMSO, g/m^2^	0.0625	0.25	0.50	0.75	1.00	2.00	3.00	4.00
Survival rate, %	100.0	100.0	83.3	50.0	33.3	33.3	16.7	0.0

### Insect breeding stock maintenance

Larvae and imagoes of *T. molitor* and imagoes of *O. sabulosum* were maintained in laboratory conditions at 23–25 °C and 40–50% relative humidity. *T. molitor* larvae were reared on pressed oat grain substrate with fresh cabbage leaves as moisture source, renewed every 5 days to prevent molding (Clopton, Janovy & Percival, 1992; Clopton & Janovy, 1993; Rybalka & Brygadyrenko, 2025). Only medium-sized larvae were selected for experiments to ensure developmental uniformity and standardized physiological responses across treatments. We tested both larval and adult stages of *T. molitor* to compare life stage sensitivity, while only adult *O. sabulosum* were used due to their annual reproductive cycle with new generation emergence only at the end of August and prolonged underground larval development phases (Brygadyrenko & Nazimov, 2015). Adult beetles (*T. molitor* and *O. sabulosum* imagoes) were maintained on natural litter substrate collected from reference steppe ecosystems where these species occur naturally.

### Lethal dose (LC_50_)

All reagents (Table 3) came from Sigma-Aldrich Chemie GmbH (Taufkirchen, Germany, 2023).

**Table 3:** Decomposition products of rocket fuel and explosives used in the experiment.

Common name	IUPAC name	CAS number	Structural formula	Melting point, °C	Boiling point, °C	Solubility in water, mg/L	Solubility in ethyl alcohol, mg/L	Solubility in DMSO, mg/L	Sources of origin
Anthracene	Anthracene	120-12-7	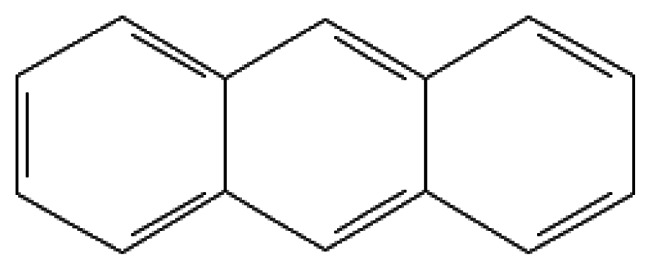	342.0	218.0	4.4*10^−5^	1.9	high	Used as a precursor for the synthesis of dyes and studied as a potential explosive
Fluorene	9H-Fluorene	86-73-7	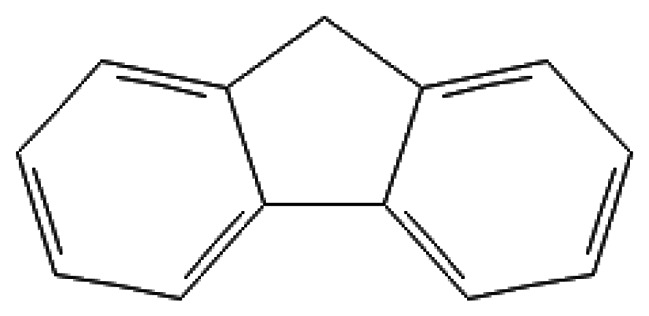	294.0	114.7	1.9*10^−3^	1.50	75.00	Used in the production of materials with high energy density; can be a component of solvents or ignition systems
Phenanthrene	Phenanthrene	85-01-8	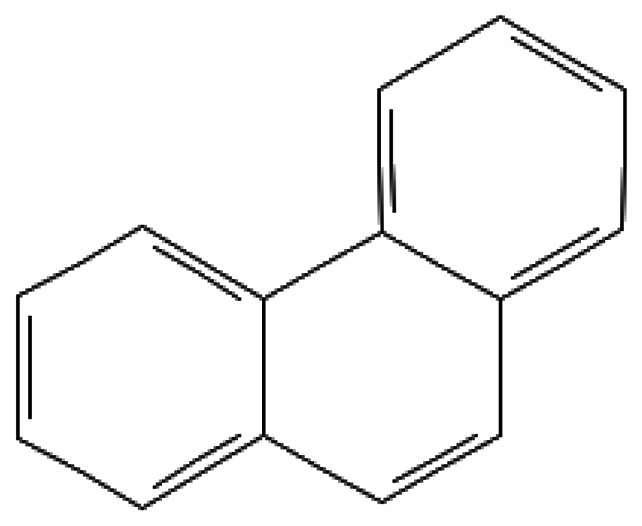	338.4	99.0	1.2*10^−3^	2.00	100.00	Polycyclic aromatic hydrocarbon; not an explosive, but may be produced during combustion or explosion of fuel
Naphthalene	Naphthalene	91-20-3	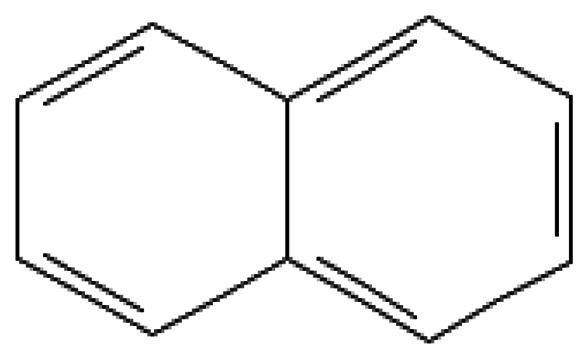	218.0	80.2	3.1*10^−2^	95.00	250.00	Historically used in military pyrotechnics; may be a precursor of explosive derivatives
*α*-Nitronaphthalene	1-Nitronaphthalene	86-57-7	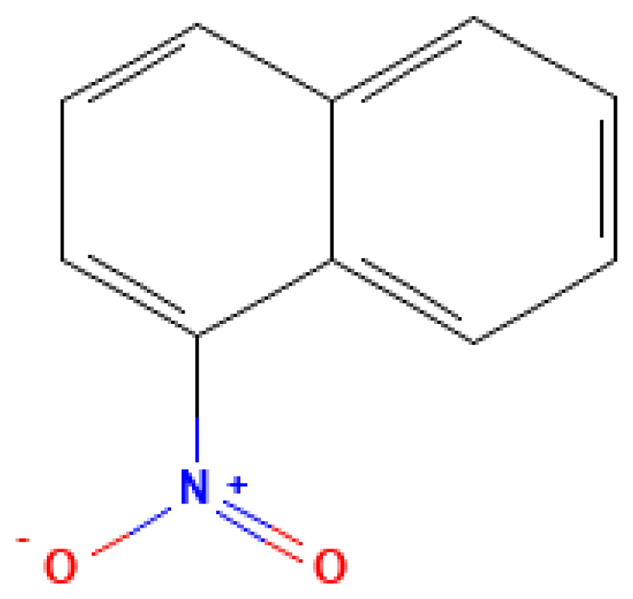	304.0	61.0	4.0*10^−3^	2.00	100.00	Various nitronaphthalenes –explosive compounds or their precursors
1-Nitroso-2-naphthol	1-Nitroso-2-naphthol	131-91-9	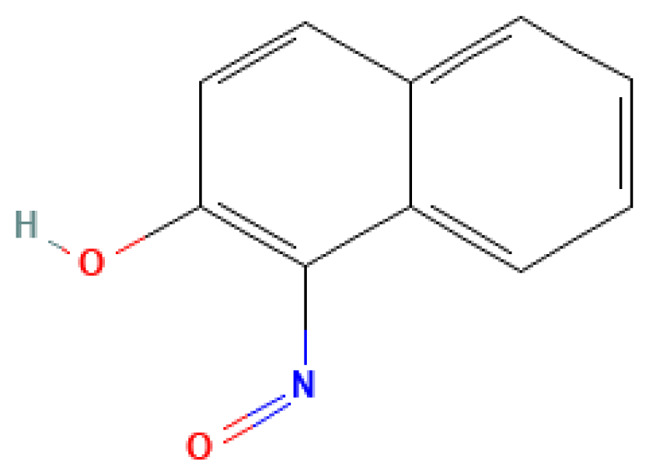	N/A	N/A	0.10	10.00	50.00	Nitroso compound of naphthalene; may be a decomposition product of explosives
N-Phenyl-1-naphthylamine	N-Phenylnaphthalen- 1-amine	90-30-2	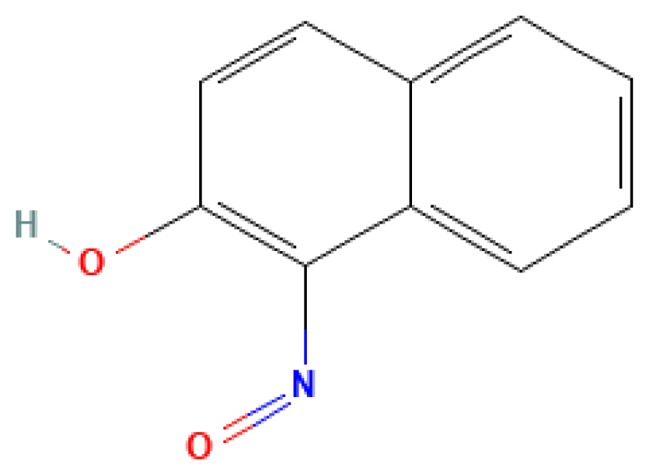	335.0	61.0	2.0*10^−3^	1.00	50.00	Aromatic amine compound that can be used in rocket propellants or colorants
p-Nitroaniline	4-Nitroaniline	100-01-6	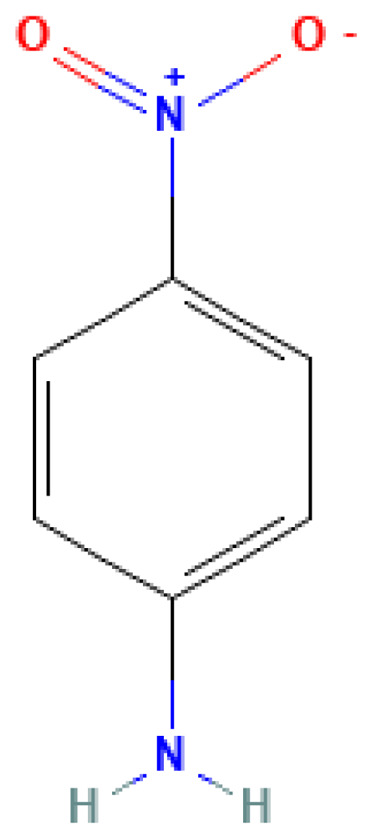	332.0	146.0	1.70	10.00	50.00	An important intermediate in the production of explosives (nitroaromatic compounds)
m-Nitroaniline	3-Nitroaniline	99-09-2	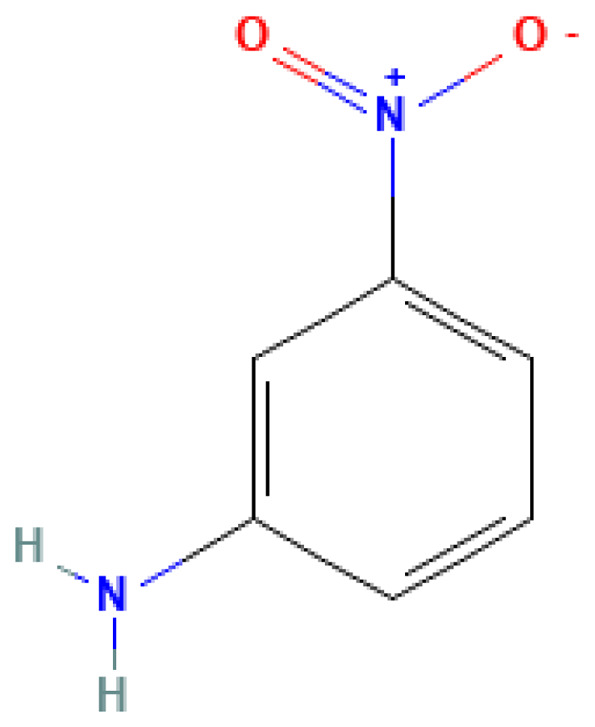	306.0	114.0	1.50	9.50	47.50	Intermediate for the synthesis of dyes and explosives
4-Methoxy-2-nitroaniline	4-Methoxy-2-nitroaniline	96-96-8	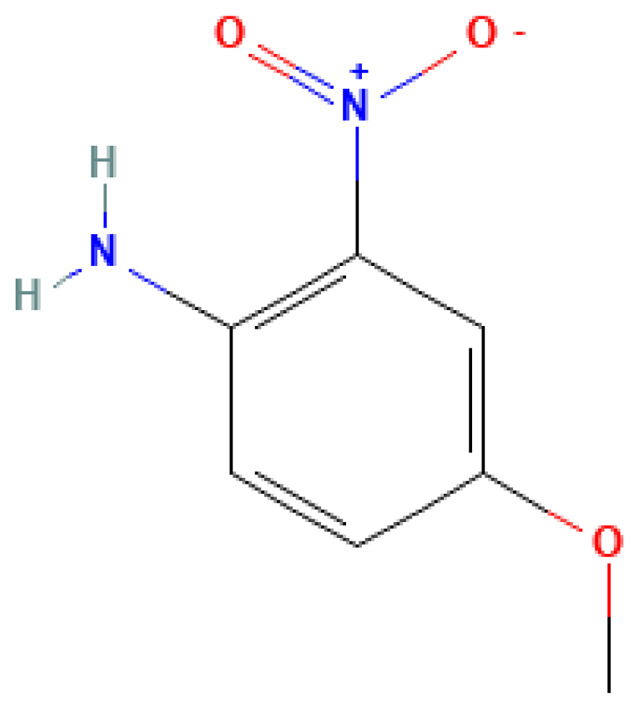	N/A	N/A	0.85	8.60	43.00	Nitroaniline derivative; potential explosive intermediate
4-Ethoxy-2-nitroaniline	4-Ethoxy-2-nitroaniline	616-86-4	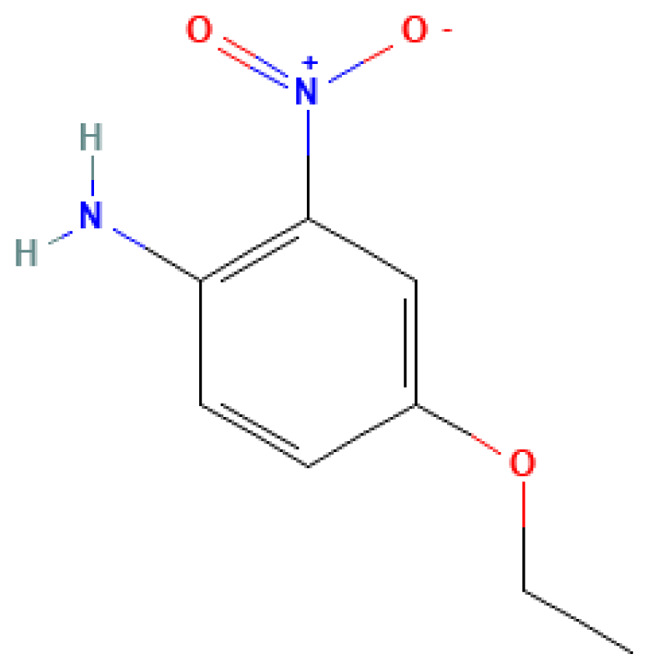	N/A	N/A	0.70	8.20	41.00	Aromatic nitro compound, possible precursor in explosives
2-Bromo-4-nitroaniline	2-Bromo-4-nitroaniline	13296-94-1	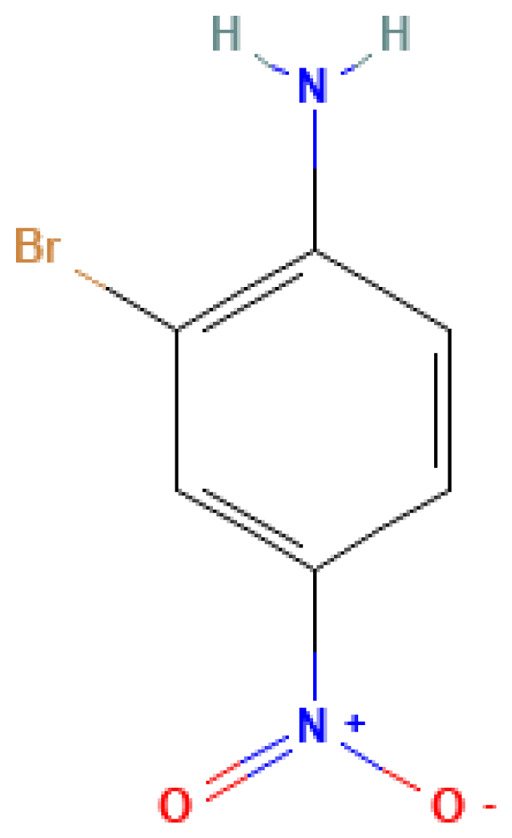	N/A	N/A	0.80	8.50	42.50	Can be used in the synthesis of nitroaromatic compounds or as a component of ammunition
2,4-Dinitroaniline	2,4-Dinitroaniline	97-02-9	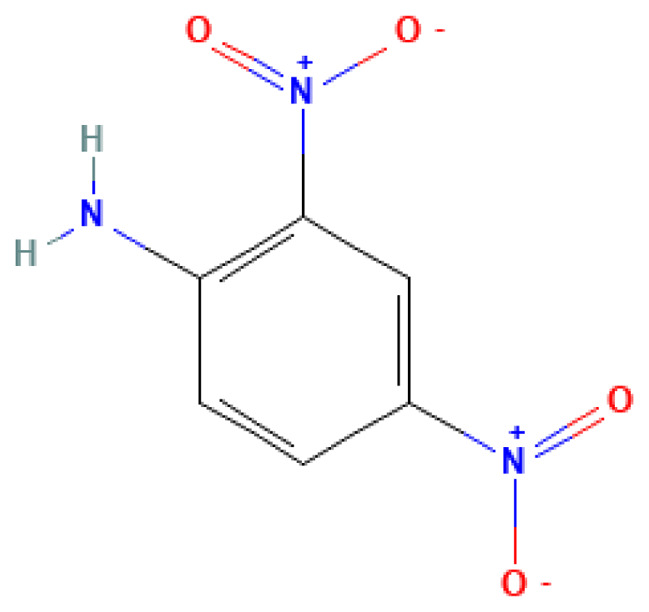	56.7	188.0	0.06	6.00	30.00	Used in the production of explosives and as a component for solid fuels
2-Methoxy-5-nitroaniline	2-Methoxy-5-nitroaniline	99-59-2	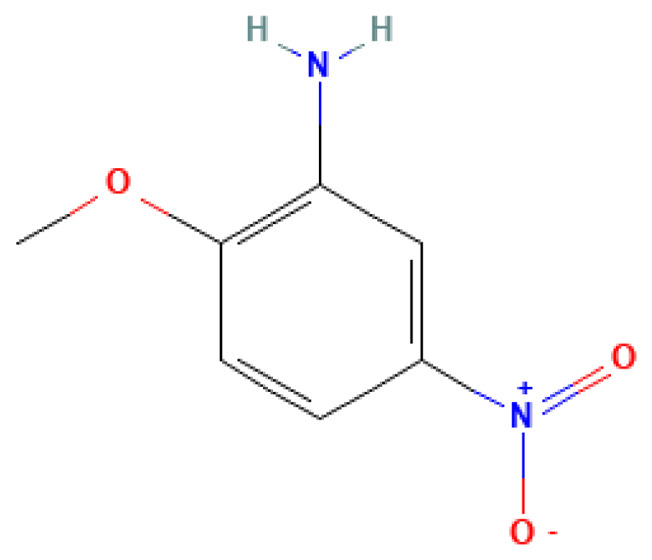	N/A	118.0	0.90	8.80	44.00	May be an intermediate in the production of explosives
4,5-Dibromo-2-nitroaniline	4,5-Dibromo-2-nitroaniline	75293-97-9	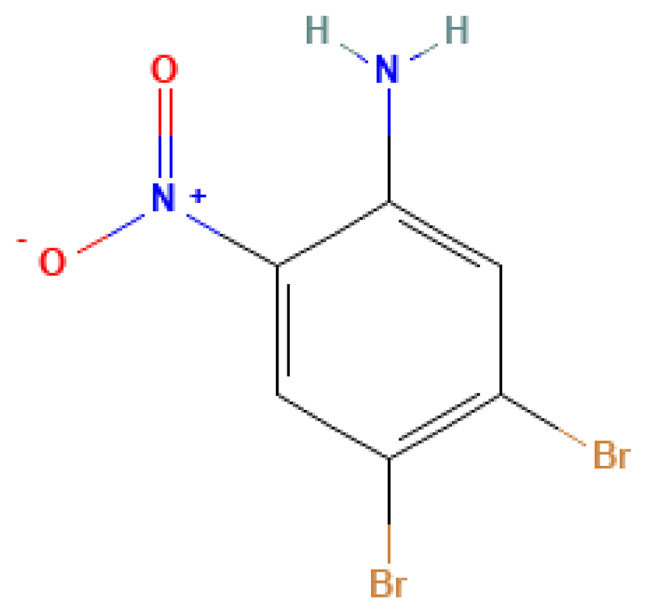	N/A	N/A	0.03	2.00	10.00	Halogenated nitro compound with potential use in explosive systems or pyrotechnics
4,5-Dimethyl-2-nitroaniline	4,5-Dimethyl-2-nitroaniline	6972-71-0	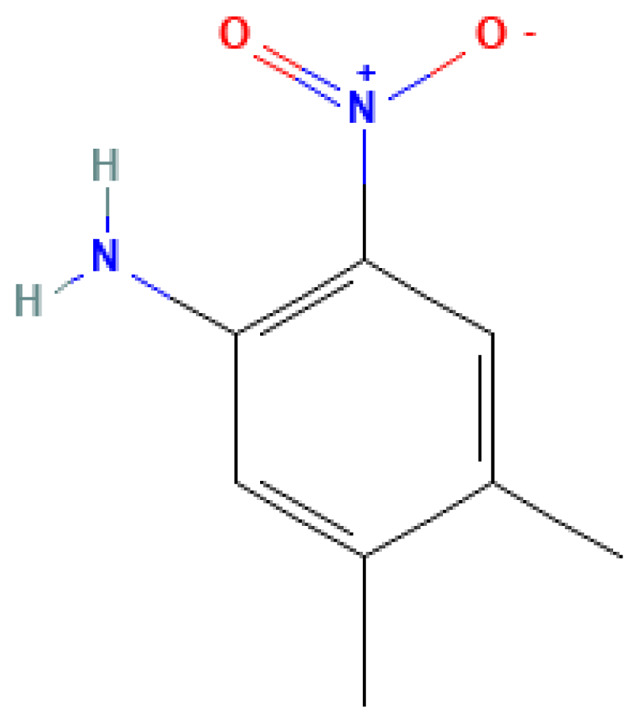	N/A	N/A	0.90	9.00	45.00	Specialized nitro compound that can be a component of energy materials
o-Nitrotoluene	2-Nitrotoluene	88-72-2	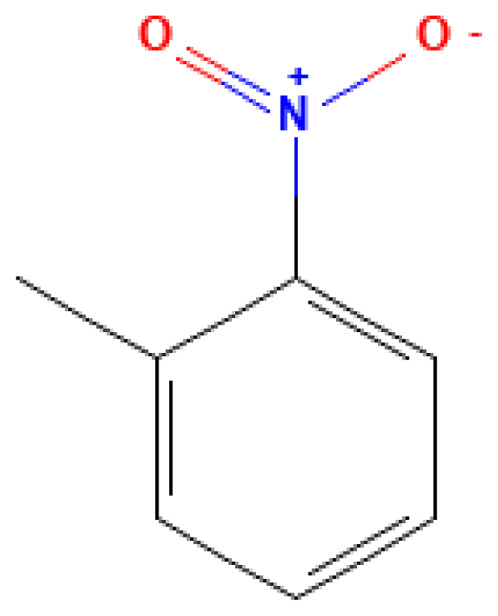	222.0	−10.0	0.10	12.00	60.00	Intermediate in the synthesis of trinitrotoluene (TNT)
2,4-DNT	2,4-Dinitrotoluene	121-14-2	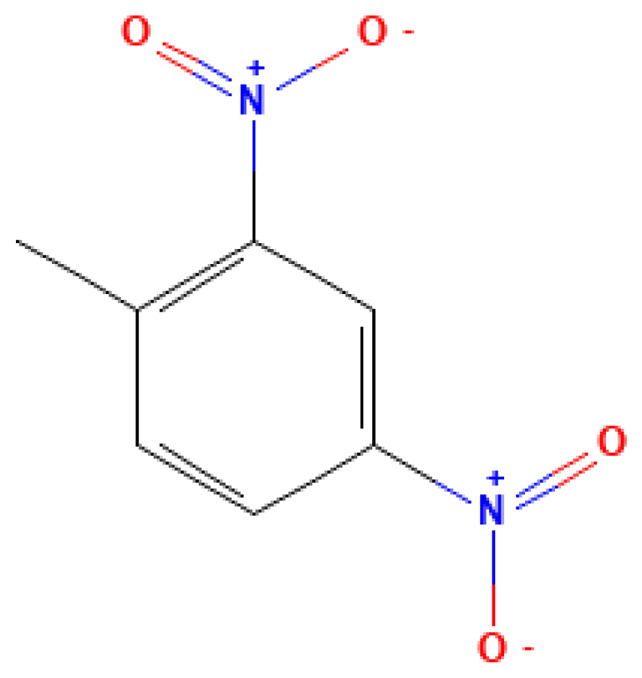	300.0	70.5	0.05	8.00	40.00	An intermediate in the production of heating oil; also used as an additive to solid fuels
1-Chloro-2,4-dinitrobenzene	1-Chloro-2,4-dinitrobenzene	97-00-7	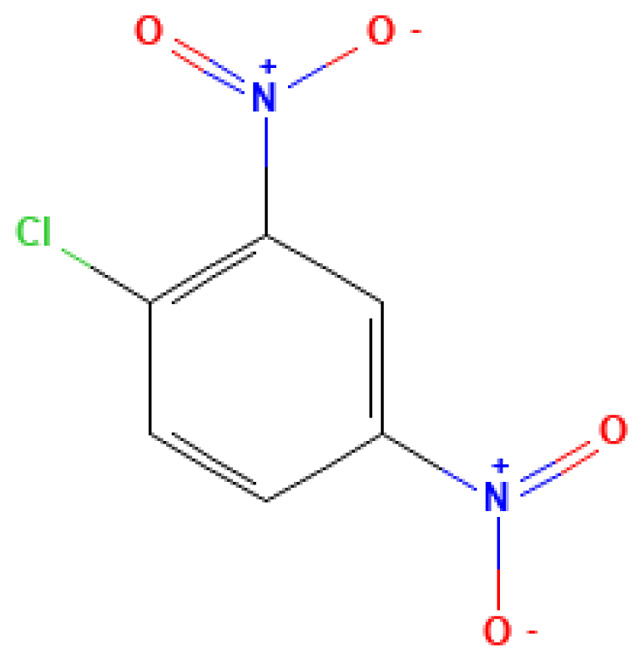	315.0	54.0	0.01	0.30	15.00	TNT derivative; used in the synthesis of explosives and as a contamination marker
p-Chloronitrobenzene	1-Chloro-4-nitrobenzene	100-00-5	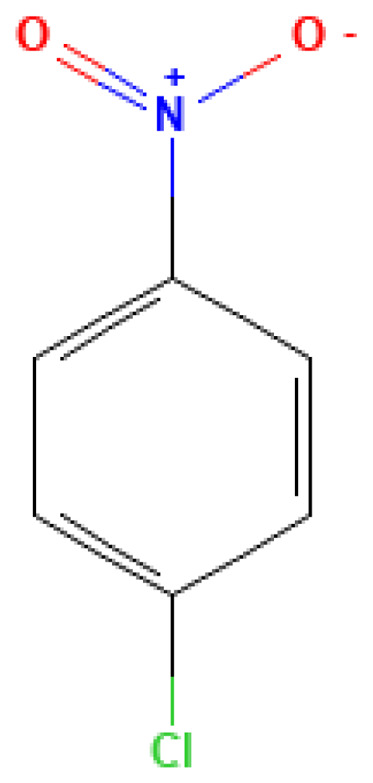	242.0	84.0	0.02	0.40	20.00	Derivative of nitrobenzene; possible presence in ammunition production wastes
p-Nitrophenol	4-Nitrophenol	100-02-7	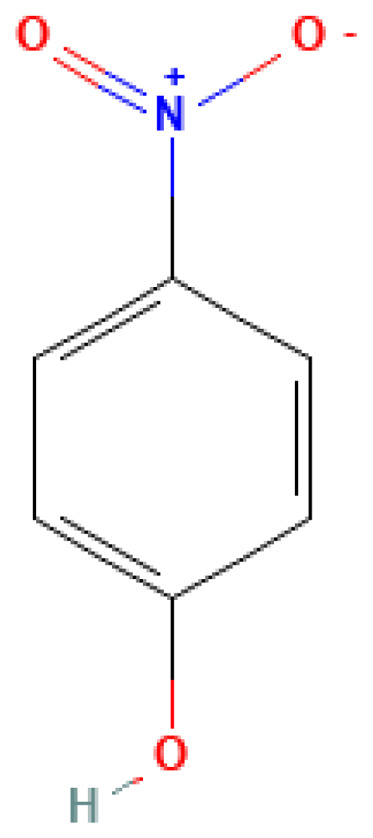	279.0	113.8	11.60	100.00	high	One of the main degradation products of TNT; widely detected in explosive contaminated soil and water
2-Hydroxy-5-nitrobenzaldehyde	2-Hydroxy-5-nitrobenzaldehyde	97-51-8	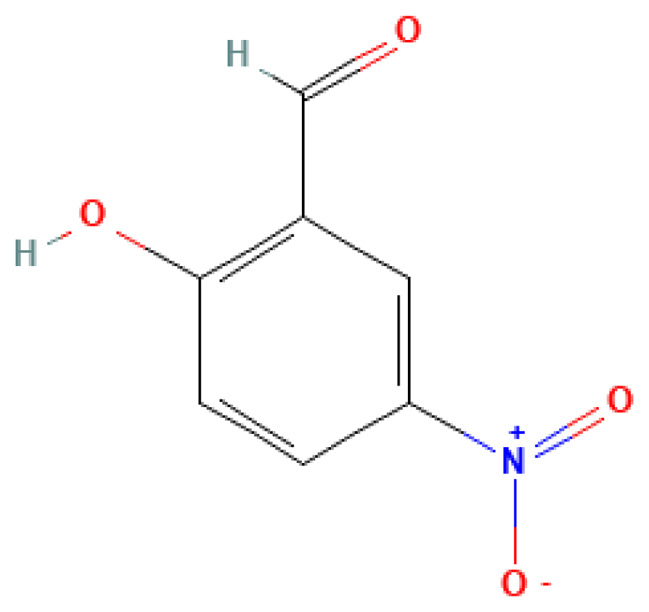	N/A	N/A	0.50	7.00	35.00	May be a decomposition product of nitro compounds or a derivative of explosives
HCB	Hexachlorobenzene	118-74-1	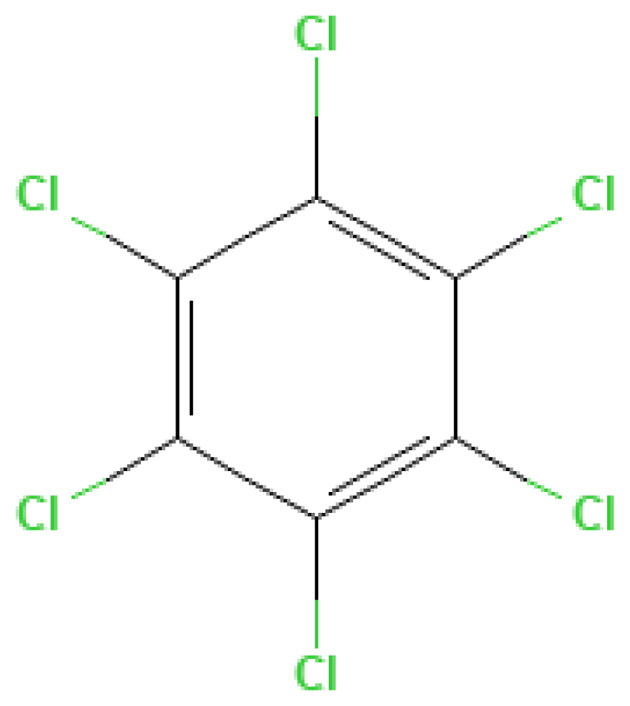	325.0	228.8	4.7*10^−6^	poorly soluble	high	Used as insecticide and inflammatory agent; toxic by-product of explosives production, persistent in the environment
TNT	2,4,6-Trinitrotoluene	118-96-7	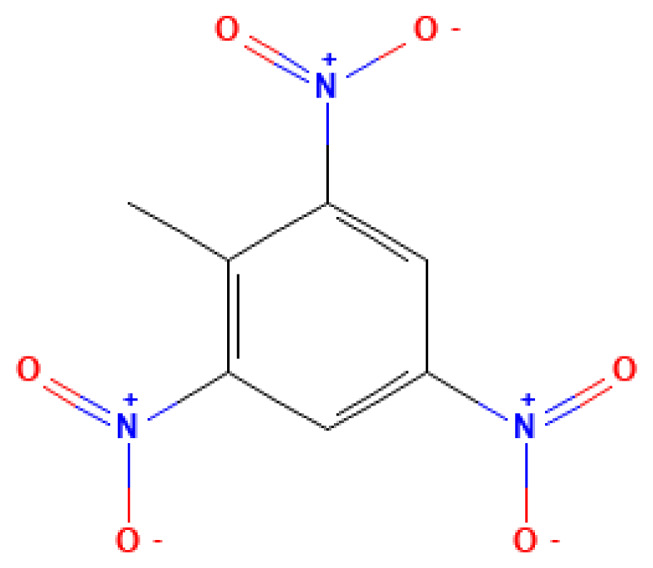	240.0	80.1	1.3*10^−2^	0.19	10.00	Classic secondary explosive; widely used in military ammunition of various types
2-Bromo-1,3,5- trinitrobenzene	2-Bromo-1,3,5- trinitrobenzene	4185-53-9	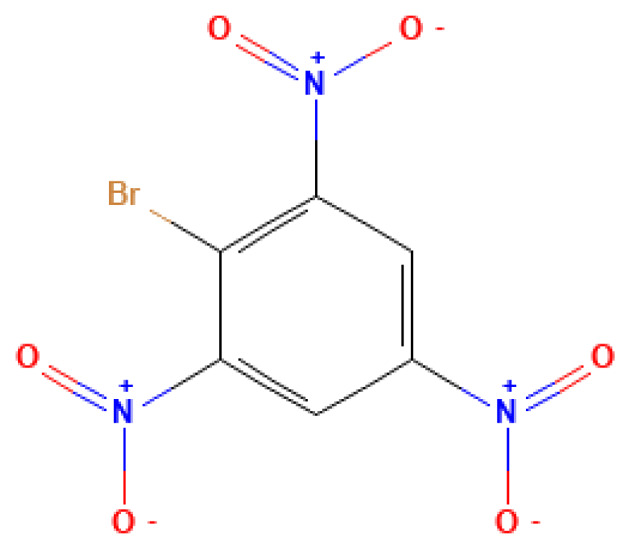	N/A	N/A	2.0*10^−3^	0.20	1.00	Close to trinitrobenzene, used as a high-energy explosive
Pentachloropyridine	Pentachloropyridine	2176-62-7	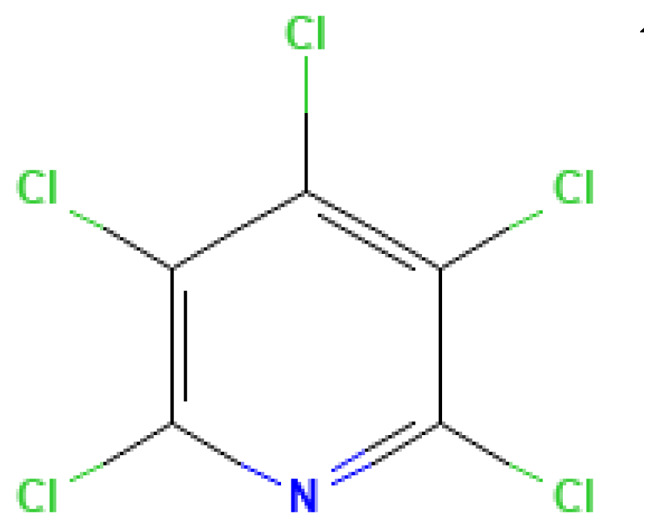	280.0	125.5	5.0*10^−3^	0.50	2.50	May be a by-product of explosives degradation or an inhibitor in fuel mixtures
Hydrazine hydrate	Hydrazine monohydrate	10217-52-4	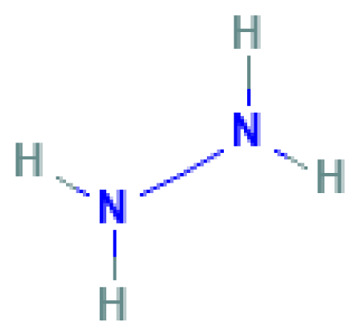	N/A	N/A	fully miscible	fully miscible	fully miscible	High-energy liquid propellant; also used in explosive systems as a reducer or hypergolic
DNPH	2,4-Dinitrophenylhydrazine	119-26-6	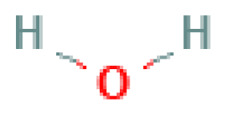	N/A	N/A	0.01	5.00	25.00	Compound with a high nitrogen content, close to sensitive explosives such as picrylhydrazine
			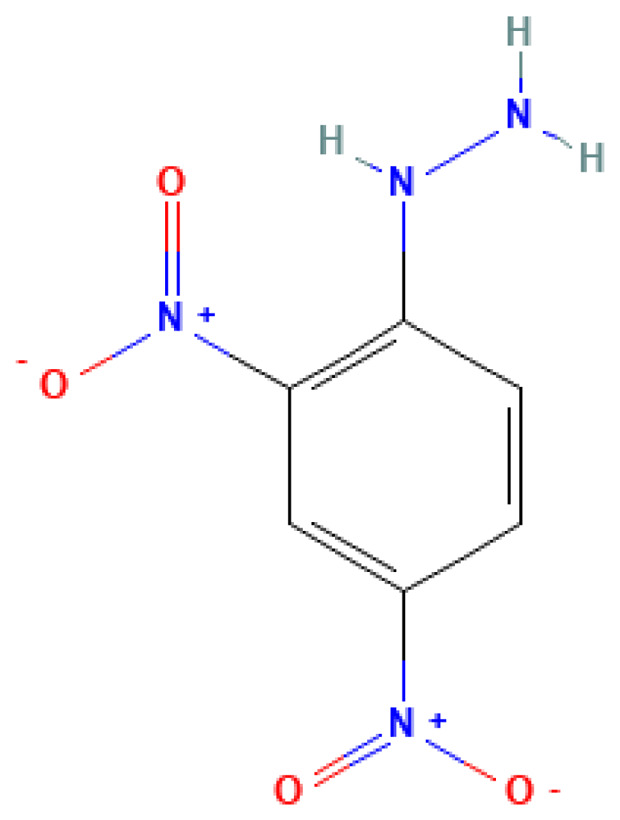						
Dimethyl 3-nitrophthalate	Dimethyl 3-nitrophthalate	13365-26-9	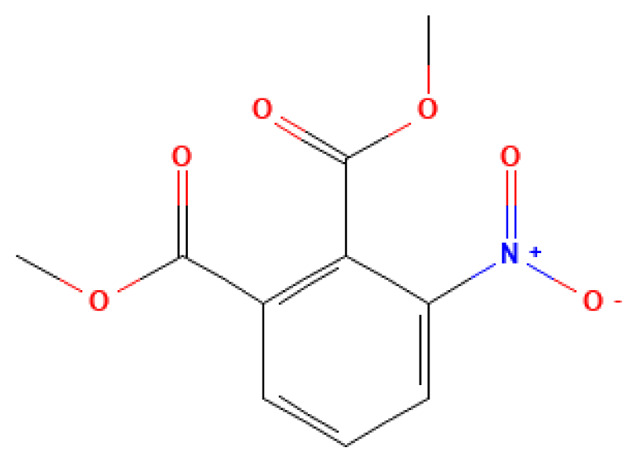	N/A	N/A	0.05	5.00	25.00	Phthalate esters with nitro groups can be used as plasticizers in solid fuels

**Notes.**

Most data are taken from Sunahara et al. (2009), as well as from publicly available chemical databases; the order of substances is given in accordance with the International Union of Pure and Applied Chemistry (IUPAC) classification principles—by type of structural core, number of rings, presence of heteroatoms and functional groups (nitro-, amino-, hydrazine-, halogenated derivatives, *etc*.); within each group, compounds are ordered by IUPAC names without taking into account numerical loci (positions); the abbreviation “N/A” is used to indicate missing or unavailable data.

To quantify the toxicity of each of the tested organic compounds, we sprayed the test solutions evenly into the containers with larvae and imagoes of *T. molitor* and imagoes of *O. sabulosum* at the rate of 0.36 mL per 10 ×10 cm container. Each container contained eight insects. Control groups received only the corresponding solvent without the active ingredient in the same amount (Martínez et al., 2018; Brai et al., 2023).

The method of surface application, by spraying, is an alternative approach used in ecology that allows modeling the actual conditions of exposure of animals to toxic substances. In this method, the substance is applied (sprayed) directly to the surface of the insect’s body, which allows the assessment of contact toxicity. This approach is especially useful for assessing the toxicity of pesticides and other chemicals that act in a contact manner (through the cuticle) (Burgess, King & Geden, 2020).

The surface application method is particularly relevant for assessing the toxicity of combustion products of explosives and rocket fuel, as these substances are often distributed as aerosols or fine particles that settle over large areas. According to a study by Zeng et al. (2024), explosive spraying is a common method for generating large volumes of vapor clouds. During the explosion, the shell of the explosive device is destroyed, and fuel droplets or explosive particles are dispersed in the air, forming a potentially explosive vapor cloud or aerosol (Burgess, King & Geden, 2020).

Under such conditions, the surface application method allows modeling of the real ways in which toxic substances affect organisms, through contact with the surface of the organism under study, the respiratory tract, or ingestion (Paramasivam & Selvi, 2017). The surface application method allows one to take into account factors such as the amount of substance per unit area, which is important for risk assessment in real-world conditions, compared to traditional methods that determine the dose per unit body weight (Dash et al., 2020). Taking the following into account, to assess the toxicity of explosive and rocket propellant combustion products, the surface application method is more consistent with the actual conditions of exposure to animals and provides more accurate data on the risks to organisms in the natural environment.

### Data analysis

We determined the median lethal concentration (LC_50_) using the regression analysis method. All calculations were performed in the R software environment (version 4.4.2; R Core Team, 2024) using the RStudio environment (version 2024.12.0+467; RStudo Team, 2024). The lethal dose of 50% (LC_50_) was estimated by regression analysis proposed by Finney (1947) using a probit model on a logarithmic dose scale. The two-parameter log–logistic regression function (LL.2) from the drc package (version 3.2-0) was used to build the model. The calculation of LC_50_, standard error (SE), and 95% confidence interval was performed using the delta method (Huang, 2010; Ritz et al., 2015). To check the fit and select the model, we used the *χ*^2^-Pearson (goodness-of-fit) from the drc package (Ritz et al., 2015). The results are presented in Tables 3 and 4. Data preprocessing (cleaning, formatting, merging tables, filling in missing values) was performed using the packages zoo (version 1.8-12), readr (version 2.1.5), tidyr (version 1.3.1), and dplyr (version 1.1.4).

**Table 4:** Toxicity (LD_50_) for rats and mice of the substances used in our study.

Substance name according to IUPAC	LD_50_ (rat oral), mg/kg	LD_50_ (mouse oral), mg/kg
Anthracene	>16,000	∼2,000
9H-Fluorene	5,628	N/A
Phenanthrene	1,800	700
Naphthalene	∼2,200	533
1-Nitronaphthalene	120	490
1-Nitroso-2-naphthol	500	400
N-Phenylnaphthalen-1-amine	1,625	1,231
4-Nitroaniline	∼750	810
3-Nitroaniline	535	310
4-Methoxy-2-nitroaniline	14,100	N/A
4-Ethoxy-2-nitroaniline	N/A	N/A
2-Bromo-4-nitroaniline	N/A	N/A
2,4-Dinitroaniline	285	N/A
2-Methoxy-5-nitroaniline	2,250	1,060
4,5-Dibromo-2-nitroaniline	N/A	N/A
4,5-Dimethyl-2-nitroaniline	N/A	N/A
2-Nitrotoluene	891	970
2,4-Dinitrotoluene	517	652
1-Chloro-2,4-dinitrobenzene	∼640	N/A
1-Chloro-4-nitrobenzene	∼420	∼440
4-Nitrophenol	220–620	380–470
2-Hydroxy-5-nitrobenzaldehyde	1,838	672
Hexachlorobenzene	∼3,500	∼4,000
2,4,6-Trinitrotoluene	∼800	∼660
2-Bromo-1,3,5-trinitrobenzene	N/A	N/A
Pentachloropyridine	435	N/A
Hydrazine monohydrate	129	83
2,4-Dinitrophenylhydrazine	654	N/A
Dimethyl 3-nitrophthalate	N/A	N/A

**Notes.**

See Table 2; data are based on the results of previous studies by Williams et al. (2017), Kim et al. (2019).

**Figure 1:**
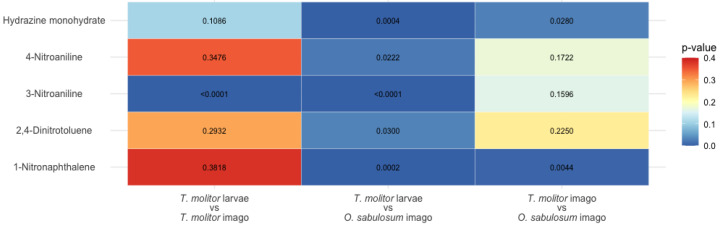
Matrix of *p*-values obtained from the bootstrap LC_50_ analysis (*n* = 10,000) between *T. molitor* larvae, *T. molitor* imagoes, *O. sabulosum* imagoes for five organic compounds. The color scale represents the *p*-value in the range of 0.0–0.4, where blue tones correspond to statistically significant differences (*p* < 0.05), and white, yellow and red tones to insignificant differences.

**Figure 2:**
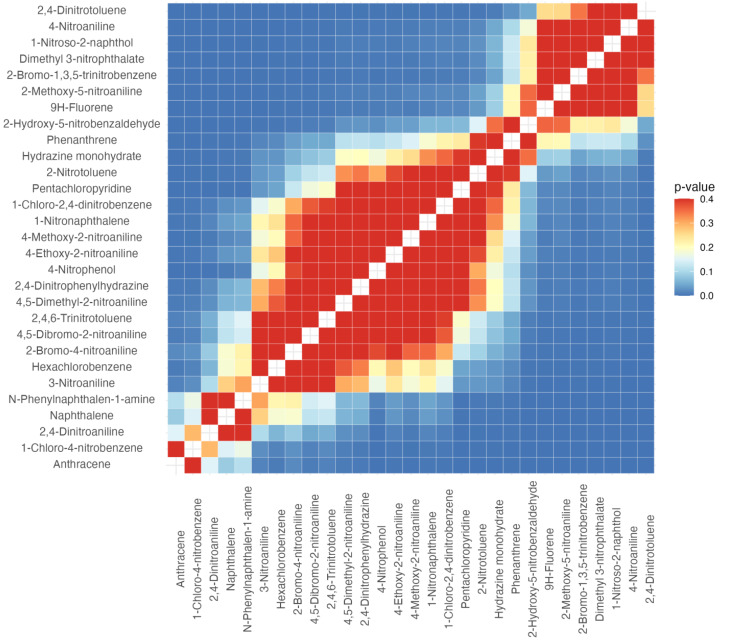
Heat map of paired *p*-values obtained from LC50 bootstrap analysis (*n* = 10,000) for 29 organic compounds tested on *T. molitor*. The color scale shows the *p*-value in the range of 0.0–0.4, where blue tones correspond to statistically significant differences (*p* < 0.05) and white yellow and red tones to insignificant differences.

To build bootstrap-distributions of LC_50_ values, *n* = 10,000 Monte Carlo simulations were performed, using a normal distribution based on the mean LC_50_ and standard error (Efron & Tibshirani, 1993). We used the resulting distributions to calculate two-sided *P*-values between pairs of models using simulated hypothesis testing (Figs. 1 and 2). For multicomponent comparisons between forms of organism within each compound, we used generation of group letters (Tables 3 and 4) using the multcompLetters function from the multcompView package (version 0.1-9) (Schmidt et al., 2015; Piepho, 2018).

We visualized the results using the ggplot2 package (version 3.5.1). To calculate Spearman’s correlation coefficients between the toxicity of substances in different model organisms (larvae and imagoes of *T. molitor*, imagoes of *O. sabulosum,* mice and rats), the cor() function with the method parameter = “spearman” was used.

## Results

The toxicity of 29 chemical compounds to larvae and imagoes of *T. molitor* and imagoes of *O. sabulosum* is presented in Tables S1–S3, which served as the basis for the probit analysis. Based on these data, we identified the limits of toxicity of each compound, which allows us to compare their acute toxicity between species and developmental stages of beetles (Tables 5 and 6). We found no deaths of larvae and imagoes of *T. molitor* and imagoes of *O. sabulosum* in the control groups.

**Table 5:** LC50 ± SE, 95% confidence intervals, dose-response curve slope and results of *χ*^2^ Pearson model checking for the tested organic compounds at the stages o f larva of *T. molitor, imago* of *T. molitor* and imago of *O. sabulosum*.

Compound name	Species and developmental stage	LC_50_± SE	Confidence interval, lower limit	Confidence interval, upper limit	Slope	Standard error of slope	Pearson’s *χ*^2^	*P*-value
3-Nitroaniline	*T. molitor* larva	0.0204 ± 0.0028^a^	0.00325	0.01407	−2.75	0.68	2.945	0.983
	*T. molitor* imago	0.0432 ± 0.0057^b^	0.00700	0.02950	−1.89	0.40	6.446	0.776
	*O. sabulosum* imago	0.0577 ± 0.0085^b^	0.00826	0.04146	−2.16	0.45	2.631	0.989
1-Nitronaphthalene	*T. molitor* larva	0.0275 ± 0.0049^a^	0.00275	0.02207	−4.95	1.46	1.011	1.000
	*T. molitor* imago	0.0333 ± 0.0046^a^	0.00514	0.02327	−2.23	0.49	3.099	0.979
	*O. sabulosum* imago	0.0592 ± 0.0075^b^	0.01006	0.03944	−1.65	0.35	4.080	0.944
Hydrazine monohydrate	*T. molitor* larva	0.0351 ± 0.0058^a^	0.00424	0.02680	−3.24	0.76	7.568	0.671
	*T. molitor* imago	0.0523 ± 0.0090^a^	0.00573	0.04108	−3.53	0.79	1.267	0.999
	*O. sabulosum* imago	0.0851 ± 0.0124^b^	0.01229	0.06103	−2.08	0.45	3.454	0.969
4-Nitroaniline	*T. molitor* larva	0.0635 ± 0.0128^a^	0.00455	0.05454	−7.66	2.40	1.599	0.996
	*T. molitor* imago	0.0815 ± 0.0144^ab^	0.00846	0.06491	−3.63	0.83	5.411	0.862
	*O. sabulosum* imago	0.1132 ± 0.0181^b^	0.01428	0.08518	−2.65	0.59	5.040	0.888
2,4-Dinitrotoluene	*T. molitor* larva	0.0757 ± 0.0145^a^	0.00640	0.06319	−5.21	1.39	1.908	0.997
	*T. molitor* imago	0.0994 ± 0.0171^ab^	0.01092	0.07796	−3.31	0.73	2.080	0.996
	*O. sabulosum* imago	0.1329 ± 0.0217^b^	0.01617	0.10124	−2.88	0.67	3.786	0.956

**Notes.**

Different letters in the LC50 ± SE column for the same substance indicate statistically significant differences between models based on bootstrap comparison (*p* < 0.05) after Schmidt et al. (2015) and Piepho (2018).

**Table 6:** LC50 ± SE, 95% confidence intervals, dose-response curve slope and results of *χ*^2^-Pearson model check for 29 organic compounds tested on *T. molitor* larvae.

Compound name	LC_50_ ± SE, g/m^2^	Confidence interval, lower limit	Confidence interval, upper limit	Slope of the dose– response curve	Standard error of the slope	Pearson’s *χ*^2^	*p*
Anthracene	0.0130 ± 0.0011^a^	0.00293	0.00728	−1.98	0.47	5.462	0.858
1-Chloro-4-nitrobenzene	0.0136 ± 0.0011^a^	0.00312	0.00753	−1.86	0.42	3.914	0.951
2,4-Dinitroaniline	0.0154 ± 0.0013^ab^	0.00348	0.00863	−1.77	0.40	9.652	0.472
Naphthalene	0.0167 ± 0.0018^abc^	0.00331	0.01018	−2.13	0.50	3.923	0.951
N-Phenylnaphthalen-1-amine	0.0168 ± 0.0021^abc^	0.00292	0.01107	−2.69	0.68	1.121	1.000
3-Nitroaniline	0.0204 ± 0.0028^bcd^	0.00325	0.01407	−2.75	0.68	2.945	0.983
Hexachlorobenzene	0.0210 ± 0.0027^bcd^	0.00355	0.01407	−2.43	0.58	4.003	0.947
2-Bromo-4-nitroaniline	0.0220 ± 0.0036^bcde^	0.00266	0.01682	−4.22	1.23	1.146	1.000
4,5-Dibromo-2-nitroaniline	0.0229 ± 0.0036^bcde^	0.00294	0.01710	−3.67	1.00	2.731	0.987
2,4,6-Trinitrotoluene	0.0231 ± 0.0038^bcde^	0.00281	0.01760	−4.00	1.13	1.827	0.998
4,5-Dimethyl-2-nitroaniline	0.0256 ± 0.0043^cdef^	0.00302	0.01969	−3.92	1.07	1.706	0.998
2,4-Dinitrophenylhydrazine	0.0259 ± 0.0044^cdef^	0.00288	0.02022	−4.30	1.22	1.968	0.997
4-Nitrophenol	0.0262 ± 0.0031^def^	0.00472	0.01693	−1.93	0.42	2.868	0.984
4-Ethoxy-2-nitroaniline	0.0264 ± 0.0041^def^	0.00342	0.01966	−3.29	0.83	2.951	0.983
4-Methoxy-2-nitroaniline	0.0270 ± 0.0040^def^	0.00385	0.01946	−2.78	0.66	5.194	0.878
1-Nitronaphthalene	0.0275 ± 0.0049^defg^	0.00275	0.02207	−4.95	1.46	1.011	1.000
1-Chloro-2,4-dinitrobenzene	0.0283 ± 0.0048^defg^	0.00322	0.02195	−3.91	1.04	1.810	0.998
Pentachloropyridine	0.0295 ± 0.0033^efg^	0.00565	0.01840	−1.66	0.36	3.035	0.981
2-Nitrotoluene	0.0318 ± 0.0044^efg^	0.00492	0.02218	−2.26	0.50	5.290	0.871
Hydrazine monohydrate	0.0351 ± 0.0058^efgh^	0.00424	0.02680	−3.24	0.76	7.568	0.671
Phenanthrene	0.0392 ± 0.0074^fghi^	0.00344	0.03248	−5.57	1.56	0.810	1.000
2-Hydroxy-5-nitrobenzaldehyde	0.0436 ± 0.0071^ghi^	0.00533	0.03318	−3.00	0.66	4.017	0.947
9H-Fluorene	0.0553 ± 0.0103^hij^	0.00506	0.04540	−5.05	1.27	2.255	0.994
2-Methoxy-5-nitroaniline	0.0553 ± 0.0103^hij^	0.00506	0.04540	−5.05	1.27	2.255	0.994
2-Bromo-1,3,5-trinitrobenzene	0.0592 ± 0.0103^ij^	0.00638	0.04673	−3.55	0.80	1.513	0.999
Dimethyl 3-nitrophthalate	0.0604 ± 0.0116^hij^	0.00505	0.05050	−5.86	1.59	1.261	1.000
1-Nitroso-2-naphthol	0.0614 ± 0.0127^hij^	0.00398	0.05363	−9.36	3.32	0.584	1.000
4-Nitroaniline	0.0635 ± 0.0128^ij^	0.00455	0.05454	−7.66	2.40	1.599	0.996
2,4-Dinitrotoluene	0.0757 ± 0.0145^j^	0.00640	0.06319	−5.21	1.39	1.908	0.997

**Notes.**

Different letters in the LC50 ± SE column for each substance indicate statistically significant differences between the models based on bootstrap comparison (*p* < 0.05) according to Schmidt et al. (2015) and Piepho (2018).

At the larval stage, *T. molitor* showed the highest sensitivity to the toxic effects of the studied substances of all model organisms involved in the experiment. Analysis revealed the lowest value of LC_50_ for 3-nitroaniline (0.0204 ± 0.0028 g/m^2^). *O. sabulosum* imagoes showed significantly higher resistance, as reflected in the highest LC_50_ value (0.1329 ± 0.0217 g/m^2^) for 2,4-dinitrotoluene. The slopes of the dose–response curves ranged from −1.65 (*O. sabulosum* imago, 1-nitronaphthalene) to −7.66 (*T. molitor* larva, 4-nitroaniline), indicating a gradual (for 1-nitronaphthalene) and sharp (for 4-nitroaniline) increase in mortality with increasing dose (Table 5). All models showed a high value of reliability for the Pearson’s *χ*^2^ goodness-of-fit test (*p* much greater than 0.05), which confirms the validity of the choice of models and the homogeneity of the input data (mortality and dose values).

To detect differences between toxicant doses and organism type, a bootstrap analysis of 10,000 LC_50_ simulations was performed based on the mean values and their standard errors for each of the three models (*T. molitor* larvae, *T. molitor* imagoes, *O. sabulosum* imagoes). For the five substances in our experiment, we found statistically significant differences between the LC_50_ for the *T. molitor* larvae and *O. sabulosum* imago models (*p* < 0.05, middle column of Fig. 1). For larvae and imagoes of *T. molitor*, we found significant differences only for 3-nitroaniline, indicating a similar toxic effect between these organisms (first column of Fig. 1). For the pair imagoes of *T. molitor* and *O. sabulosum*, we observed significant differences in LC_50_ for hydrazine monohydrate and 1-nitronaphthalene (third column of Fig. 1).

The results of the probit analysis for the 29 organic compounds showed low variability in the mean lethal dose ± standard error (LC_50_ ± SE): from the most toxic anthracene (0.0130 ± 0.0011 g/m^2^) to approximately 6 times higher LC_5__0_ values for 2,4-dinitrotoluene (0.0757 ± 0.0145 g/m^2^), indicating low differences in the toxicity of substances to *T. molitor* larvae. The slopes of the dose–response curves ranged from −1.66 (pentachloropyridine) to −9.36 (1-nitroso-2-naphthol), which indicates a gradual (for pentachloropyridine) and sharp (for 1-nitroso-2-naphthol) increase in mortality with increasing dose. Statistical tests of model consistency (Pearson’s goodness-of-fit *χ*^2^) revealed no significant deviations between the experimental data and the curve approximation. This indicates the sufficient quality of the built models and allows the use of the obtained LC_50_ values for further comparisons of toxicity between compounds (Table 6).

**Figure 3:**
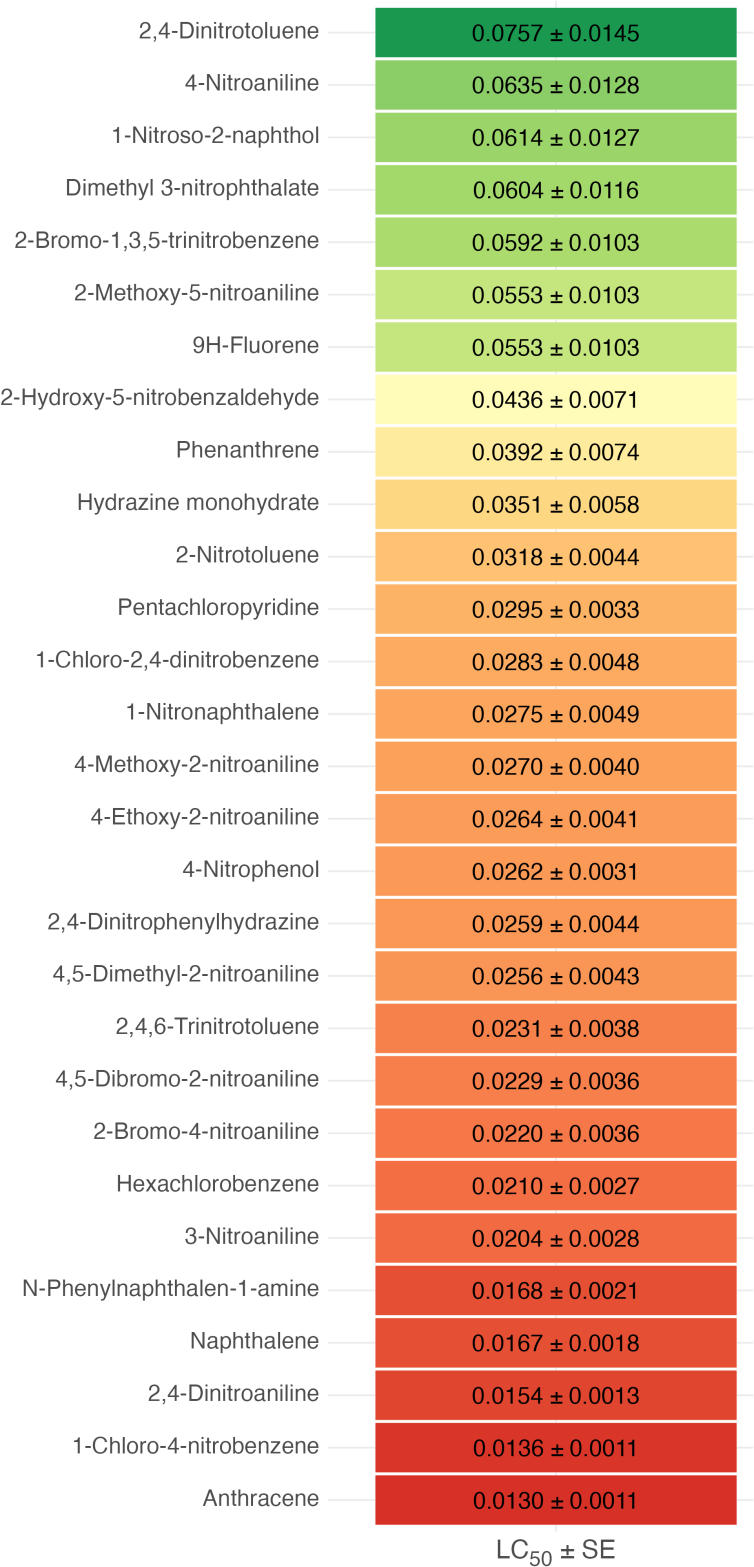
Visualization of LC_50_ values (g/m^2^) for 29 organic compounds ordered by increasing toxicity (decreasing LC_50_) tested on *Tenebrio molitor* larvae. Each line corresponds to the mean LC_50_ value with standard error (±SE); the color scale represents the toxicity gradient: red are the most toxic substances with the lowest LC_50_ values, green are less toxic substances.

The heat map reflects the ranking of the 29 studied organic compounds by increasing average lethal dose (Fig. 3), ranging from the most toxic anthracene (0.0130 ± 0.0011 g/m^2^) and 1-chloro-4-nitrobenzene (0.0154 ± 0.0013 g/m^2^) to approximately 5–6 times higher LC_5__0_ values for 2,4-dinitrotoluene (0.0757 ± 0.0145 g/m^2^), which allows us to clearly identify the substances with the highest and lowest toxic effects on *T. molitor* larvae.

## Discussion

The compounds under study are directly related to explosives. TNT is one of the most common explosives widely used in arms production in most countries of the world due to its high detonation energy, relative stability during storage, and ease of forming into ammunition (Ouyang et al., 2022). Other benzene nitro derivatives, such as 2-bromo-1,3,5-trinitrobenzene and 1-chloro-2,4-dinitrobenzene, are also used as intermediates or by-products in the manufacture of explosives, which causes their accumulation in the environment and creates additional environmental risks (Ouyang et al., 2022).

Some of the compounds studied are components of or related to rocket fuel. Hydrazine monohydrate and its derivatives, including 2,4-dinitrophenylhydrazine, are widely used in liquid propellants due to their high energy density and ability to hypergolic ignition (Barato, 2023). Hydrazine monohydrate is a basic component of monopropellants used to precisely control the motion of spacecraft, although its use requires special care due to toxicity and carcinogenicity (Huang et al., 2018).

The implementation of the 3Rs (replacement, reduction, refinement) principles formulated by Russell & Burch (1959) has become a key ethical guideline for modern toxicology. The use of invertebrates, such as larvae and imagoes of *T. molitor* and *O. sabulosum*, is fully consistent with these principles, as it allows a reduction in the use of vertebrate animals in toxicity testing. Brai et al. (2023) demonstrated the successful use of *T. molitor* as a cheap and convenient preliminary toxicity model, showing that LC_50_ values are comparable to those determined in mice and rats, while significantly reducing costs and the number of rodents used according to 3R principles.

The effectiveness of invertebrate models varies depending on the specific research context and target compounds. Studies have demonstrated both successful and unsuccessful applications across different invertebrate taxa. For example, marine bivalves have been successfully used as indicator organisms for munition compound contamination (Binder et al., 2025), while *Galleria mellonella* has shown promise as a model for studying microbial infections and toxicity screening due to its low cost, ethical advantages, and mammalian-like immune responses (Marena et al., 2025), though limitations exist for some specific applications. The choice of model organism should be guided by ecological relevance to the contaminated environment rather than universal suitability claims. In the context of explosive contamination in terrestrial ecosystems, darkling beetles (*T. molitor* and *O. sabulosum*) represent ecologically relevant species that naturally occur in areas where explosive contamination is likely to persist, making them appropriate for environmental risk assessment in accordance with the 3Rs principles.

During the analysis of publications in leading scientific databases, we identified toxicological data for some of the compounds from our study list, focusing exclusively on terrestrial invertebrates, like earthworms (*Eisenia fetida* (Savigny, 1826)) and beetles (*T. molitor*). Note that direct comparison of these values is complicated by different exposure methods: µg/L represents aqueous exposure concentrations while mg/kg represents concentrations in artificial soil, reflecting different bioavailability and exposure pathways. For *E. fetida*, the LC_50_ values for anthracene and naphthalene exceeded 1,000 µg/L, indicating its very low acute toxicity (Nam et al., 2015). In the case of 9H-fluorene, an LC_50_ of 394.1 µg/L was recorded, which also indicates limited toxicity at short-term exposure (Nam et al., 2015). Phenanthrene demonstrated moderate toxicity with an LC_50_ = 114.0 µg/L (Nam et al., 2015). For 2,4-dinitrotoluene, an LC_50_ of ≈ 668.0 mg/kg in artificial soil was determined (OECD, 2003). Neuhauser et al. (1986) mentioned significant toxicity for 4-nitrophenol: the LC_50_ in soil ∼38.0 mg/kg for 14 days, indicating a high sensitivity of *E. fetida* to this compound. For TNT, Robidoux et al. (1999) recorded the LC_50_ range from 222.4 to 364.9 mg/kg depending on the soil type, which also indicates high toxicity of TNT to earthworms.

For the rest of the studied compounds, 1-nitronaphthalene, hydrazine monohydrate, 2-bromo-4-nitroaniline, 2-bromo-1,3,5-trinitrobenzene, 2-hydroxy-5-nitrobenzaldehyde, 1-chloro-2,4-dinitrobenzene, 1-chloro-4-nitrobenzene, 4-ethoxy-2-nitroaniline, 4,5-dimethyl-2-nitroaniline, N-phenylnaphthalen-1-amine, 4-methoxy-2-nitroaniline, 2-methoxy-5-nitroaniline, 2,4-dinitroaniline, dimethyl 3-nitrophthalate, pentachloropyridine, 2,4-dinitrophenylhydrazine, 1-nitroso-2-naphthol and hexachlorobenzene, we were unable to find relevant scientific publications that would contain LC_50_ values specifically for terrestrial invertebrates. The available information relates mainly to vertebrates (rats, mice) or aquatic organisms (fish, daphnia), which makes it impossible to reliably extrapolate the results to model species such as *T. molitor*, *E. fetida* or *O. sabulosum*. The absence of such data emphasizes the urgent need for targeted toxicological studies to assess the environmental risks associated with environmental contamination by explosives and rocket fuel components, especially in the context of military operations.

Comparative toxicity data for rats and mice (Table 4) were obtained from previous studies by Williams et al. (2017), Kim et al. (2019), and with additional reference data from Sunahara et al. (2009) and publicly available chemical databases. The analysis of correlations between LC_50_ values for five biological models (*T. molitor* larvae, *T. molitor* imagoes, *O. sabulosum* imagoes, rats and mice) revealed a high degree of consistency of rank values among insects. We observed a maximum correlation between *O. sabulosum* imagoes and *T. molitor* larvae (*ρ* = 1.00), as well as a strong correlation between *T. molitor* imagoes and both other darkling beetle models (*ρ* = 0.90). This indicates a similar toxicological response and potentially similar detoxification mechanisms in invertebrates.

**Figure 4:**
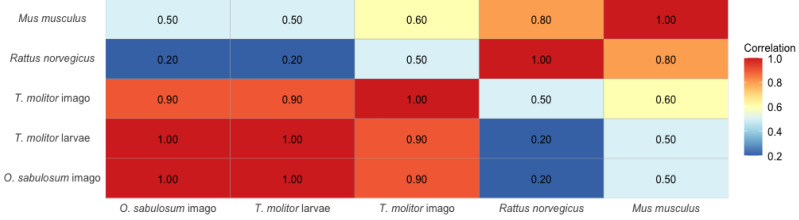
Spearman’s rank correlation matrix between LC_50_ values in *T. molitor* larvae, *T. molitor* imagoes, *O. sabulosum* imagoes, mice and rats.

In contrast, mammals (especially rats) showed low or even inverse correlations with darkling beetles, which may indicate fundamental biochemical differences in the metabolism of toxic substances (Fig. 4). Calculation pointed at correlation between rats and *T. molitor* larvae of *ρ* = 0.20, which is significantly lower than the correlation between darkling beetles. For mice, we found higher values of Spearman’s rank correlation coefficient, *ρ* = 0.60 with *T. molitor* imago and *ρ* = 0.50 with *O. sabulosum* imago.

Hodson (1985) found high correlations between intraperitoneal toxicity in fish and mammals (*r* = 0.83−0.94), but lower values for the oral route of administration (*r* = 0.59−0.66), demonstrating the influence of both the species and the route of administration.

Despite the limitations, invertebrates, like *T. molitor*, are increasingly being considered as a reliable alternative for preliminary toxicological screening due to their low cost, ethical acceptability, and comparability with mammalian data for certain groups of compounds (Brai et al., 2023). However, direct comparisons between species require caution and additional validation in larger samples.

In this study, we are unable to cover the numerous transformation products of explosion and combustion of liquid rocket fuel (Sunahara et al., 2009) due to a number of factors: limitations of the experimental base, time frame, available biomass of test organisms, and lack of toxicological data even for basic doses of many compounds.

In addition, physicochemical environmental conditions (temperature, humidity, UV radiation, soil composition) significantly affect the stability of explosive compounds, the way they degrade, and their bioavailability to organisms. For example, Cates et al. (2022) show that moisture and temperature fluctuations change the structure of soil organic matter, which in turn affects the degradation of organic contaminants. For TNT, photolysis has been shown to be largely dependent on light, moisture, and metal content in the soil (Im et al., 2015). Another limitation is the form of substances entering the natural environment - aerosols and droplets that settle unevenly over large areas. This is significantly different from classical laboratory tests in water, soil, or air. That is why the surface spray method (g/ha) that we used is a more relevant laboratory simulation of real-world conditions for terrestrial invertebrates.

Needless to say, the Spearman method, which is used as a nonparametric criterion for assessing the correlation between rank values, has certain limitations for small samples (*n* <10). In such cases, the effect of quantization of the correlation coefficient values occurs, which reduces the statistical power of the method for detecting weak relationships (Bonett & Wright, 2000; Hauke & Kossowski, 2011). This limits the interpretation of the results in our study (*n* = 5 models) and necessitates a cautious interpretation. Moreover, the limitations of comparing toxicological responses between mammals and invertebrates was demonstrated by Devillers et al. (2009): they investigated the possibility of predicting the toxicity of chemicals to rats and mice based on data on invertebrates such as *Daphnia magna* Straus, 1820 and bacteria such as *Vibrio fischeri* (Beijerinck, 1889) Leisinger & Macé (1971) using QSAR models. They found significant but not complete agreement between the interspecies models, which emphasizes the difficulty of extrapolating results to humans.

The absolute difference between these values is only 0.0627 g/m^2^, and the relative difference is about 5.8 times, which is much less than the 20–30 times difference accepted in toxicology. Munro et al. (1996) suggested using a 100-fold safety factor when extrapolating toxicity data from animals to humans, where the 100-fold factor is composed of two 10-fold components: one accounting for interspecies variation (animal-to-human differences) and another for intraspecies variation (individual sensitivity differences among humans). All the compounds we studied have a similar level of toxicity to *T. molitor* and do not form clearly differentiated groups in terms of toxicity.

## Conclusions

Given that all tested compounds showed similar toxicity levels to *T. molitor* (with only 5.8-fold difference between most and least toxic compounds, compared to typical 20–30-fold differences in toxicology), our results demonstrate that explosive and fuel compound derivatives pose a consistently moderate risk to terrestrial invertebrates. The acute toxicity values depend on both the test organism species and its weight, which requires further refinement of dose models in future experiments.

When assessing the acute toxicity of 29 chemical compounds for *T. molitor* larvae, it was found that the LC_50_ values range from 0.0130 g/m^2^ for anthracene (the most toxic compound) to 0.0757 g/m^2^ for 2,4-dinitrotoluene (the least toxic compound).

The hypothesis that it is feasible to use invertebrate models for preliminary screening of the toxicity of explosive and fuel compounds was confirmed. The method of applying the test substance by spraying and then calculating the dose in g/m^2^ allows us to adequately reproduce the exposure method typical for war zones (for example, in Ukraine), where these substances spread through the atmosphere, settling on the soil and on the surface of animals’ bodies. Based on our findings of consistent moderate toxicity across all tested compounds, we recommend: (1) prioritizing monitoring of anthracene and other aromatic compounds (LC_50_ <0.02 g/m^2^) in contaminated areas, (2) establishing surface contamination thresholds of 0.01 g/m^2^ for soil monitoring in combat zones, and (3) implementing regular biomonitoring using *T. molitor* larvae as standardized test organisms for rapid toxicity assessment of unknown explosive derivatives.

The results of our study not only fill a gap in the toxicological data on poorly studied xenobiotics, but also demonstrate the potential of using darkling beetles for risk assessment in the context of environmental safety.

##  Supplemental Information

10.7717/peerj.20427/supp-1 Supplemental Information 1 The toxicity of 29 chemical compounds to larvae and imagoes of *T. molitor* and imagoes of *O. sabulosum*

## Additional Information and Declarations

### Data Availability

The following information was supplied regarding data availability: The raw data is available in the Supplementary File.
